
JOURNAL INFORMATION
==============================
NLM Title Abbreviation: Alcohol Res Health
Journal ID: Alcohol Res Health
NLM Journal ID: 100900708
Journal ID: ARH

Alcohol Research & Health

ISSN: 1535-7414
EISSN: 1930-0573
Publisher: National Institute on Alcohol Abuse and Alcoholism

ARTICLE INFORMATION
==============================
PMCID: PMC3860465
Article ID: arh-31-4-345
Article version: 1
Subjects: Glossary

Glossary

Print publication date: 2008
Volume: 31
Issue: 4
First page: 345
Last page: 347
Copyright year: 2008
License: Unless otherwise noted in the text, all material appearing in this journal is in the public domain and may be reproduced without permission. Citation of the source is appreciated.
License URL: https://creativecommons.org/publicdomain/mark/1.0/

Comment in: Magnetic Resonance Imaging of the Living Brain 31 4 2008 362 376 Alcohol Research & Health PMC3860463 23584010
Comment in: The Molecular Basis of Tolerance 31 4 2008 298 309 Alcohol Research & Health PMC3860466 23584007
Comment in: Treatment Implications 31 4 2008 400 407 Alcohol Research & Health PMC3860468 23584013
Comment in: From Actions to Habits 31 4 2008 340 344 Alcohol Research & Health PMC3860473 23584008
Comment in: Alcohol-Related Neurodegeneration and Recovery 31 4 2008 377 388 Alcohol Research & Health PMC3860462 23584011
Comment in: How Adaptation of the Brain to Alcohol Leads to Dependence 31 4 2008 310 339 Alcohol Research & Health PMC2923844 20729980

==============================
γ-Aminobutyric acid (GABA): A neurotransmitter that in the brain acts to reduce the activity of the signal-receiving neuron.

Action potential: A rapid change in cell membrane potential (the electrical potential difference across the cell membrane) followed by a return to the resting potential. An action potential is the basis of the signal-carrying ability of nerve cells.

Adrenal glands: Small structures located on top of the kidneys that produce numerous hormones (e.g., glucocorticoids, which are important factors in the stress response).

Affinity: A measure of how easily and tightly a substance binds to a receptor.

Agonist: An agent that mimics the actions or effects of another agent (e.g., a drug that mimics the effects of a neurotransmitter).

Alcohol deprivation effect: Animal model of relapse in which animals are first chronically exposed to alcohol, followed by a period during which alcohol is withheld; if alcohol is then reintroduced, the animals consume more alcohol than before the enforced abstinence period.

Allele: One of two or more variants of a gene or other DNA sequences.

Alternative splicing: The RNA-splicing variation mechanism in which the exons of the primary gene transcript, the pre-messenger RNA (mRNA), are separated and reconnected so as to produce alternative ribonucleotide arrangements. Alternative splicing uses genetic expression to facilitate the synthesis of a greater variety of proteins.

Amygdala: Almond-shaped groups of nerve cells (i.e., neurons) located deep in the brain that are involved in the processing and memory of emotional reactions.

Anisotropic: Not having properties that are all in the same direction; showing differences in a property or effect in different directions.

Antagonist: An agent that blocks or reverses the actions or effects of another agent (e.g., a drug that blocks the effects of a neurotransmitter).

Anxiogenic: Anxiety inducing.

Anxiolytic: Anxiety reducing.

Astrocytes: Characteristic star-shaped glial cells in the brain and spinal cord that, among other functions, provide nutrients to the nervous tissue, and play a principal role in repair and scarring process of the brain and spinal cord.

Axon: The long, thin fiber protruding from a nerve cell that carries integrated nerve signals in the form of electrical signals to other nerve cells.

Basal ganglia: A group of nuclei in the brain interconnected with the cerebral cortex, thalamus, and brainstem. Mammalian basal ganglia are associated with a variety of functions: motor control, cognition, emotions, and learning.

Cation: Atom or molecule carrying a positive electrical charge.

Caudate nucleus: A nucleus located within the basal ganglia of the brains of many animal species. It is involved with control of voluntary movement, and is an important part of the brain’s learning and memory system.

Cerebellum: A region of the brain involved in the coordination of movement.

Cerebrospinal fluid: Fluid that surrounds the brain and spinal cord.

Cholinergic: Related to the neurotransmitter acetylcholine.

Chromatin: The combination of DNA, RNA, and protein that makes up chromosomes. It is formed by the DNA wrapped around histones.

Chromatin remodeling: An epigenetic mechanism, in which the chromatin structure is dynamically regulated either locally (gene expression regulation) or globally (chromosome segregation).

Cortico-basal ganglia networks: See basal ganglia.

Cortico-mesolimbic dopaminergic pathway: See mesolimbic dopamine system.

Corpus callosum: The largest connective pathway in the human brain. It is made of nerve fibers that connect the left and right sides (hemispheres) of the brain.

Corticotrophin-releasing factor (CRF): A hormone produced in response to stress that helps mediate the stress response; is produced mainly in the hypothalamus but also can be produced in other brain regions.

Cyclic AMP (cAMP): Second messenger molecule whose production by enzymes called adenylyl cyclases is regulated through receptor activation of G-proteins, and which itself activates certain protein kinases.

Dentate granule cells: Tiny neurons (i.e., granule cells) that are around 10 micrometers in diameter found in the dentate gyrus.

Dentate gyrus: Part of the hippocampus that contributes to new memories as well as other functional roles; one of a few brain structures with high rates of neurogenesis in adult humans.

DNA methylation: Epigenetic mechanism of regulation of gene expression, in which a strand of DNA is modified by addition of a methyl group (CH3) to any cytosine located directly before a guanine.

Dopamine: Neurotransmitter that is involved, among other functions, in controlling behavior and cognition, motor activity, motivation and reward, mood, attention, and learning. Dopamine-producing nerve cells are located primarily in the ventral tegmental area (VTA) and in regions of the hypothalamus.

Dopaminergic: Referring to nerve cells that use dopamine as a neurotransmitter.

Endogenous opioids: A group of small molecules, such as endorphins and enkephalins, that are naturally produced in the body and which have similar effects as the opiates (e.g., morphine and heroin); endogenous opioids modulate the actions of various neurotransmitters.

Entorhinal cortex: An important memory center in the brain that generates the main input to the hippocampus and is responsible for the preprocessing (familiarity) of the input signals.

Epigenetic: Heritable changes in phenotype or gene expression caused by mechanisms other than changes in the underlying DNA sequence.

Excitatory neurotransmitter: Any neurotransmitter that in the brain acts to enhance the activity of the signal-receiving neuron.

Exon: The sequences of the primary RNA transcript (or the DNA that encodes them) that exit the nucleus as part of a messenger RNA (mRNA) molecule.

GABAergic: Referring to neurons that use γ-aminobutyric acid (GABA) as a neurotransmitter.

Gene expression: The process by which the genetic information encoded in the DNA is converted into a protein product.

Gene promoter region: A region of DNA that facilitates the transcription of a particular gene.

Genotype: The complete genetic makeup of an organism.

Genu: The anterior end of the corpus callosum.

Glial cell: Cells that provide support and protection for neurons.

Glutamate: A neurotransmitter that enhances the activity of the signal-receiving neuron; increases in glutamate activity are probably involved in some manifestations of alcohol withdrawal.

Glutamatergic: Referring to nerve cells that use glutamate as a neurotransmitter.

G-protein: Regulatory molecules located in the cell membrane or inside the cell whose actions are instigated by neurotransmitters. Several types of G-proteins exist, including stimulatory G-proteins that enhance the activities of other enzymes and inhibitory G-proteins that inhibit the activities of other enzymes.

Hippocampus: A curved ridge found within the cerebral hemisphere that functions in consolidation of new memories; also thought to play a role in alcohol withdrawal seizures.

Histones: Protein structures around which DNA strands are wrapped.

Hyperexcitability: Excessive activity of a nerve cell or neural system resulting from excessive responsiveness to excitatory neurotransmitters or reduced responsiveness to inhibitory neurotransmitters.

In vitro: Literally translated as “in glass;” refers to experiments conducted with isolated cells or tissues or with cells or tissues grown in culture dishes.

In vivo: Refers to experiments conducted in an intact animal.

Inhibitory neurotransmitter: Any neurotransmitter that in the brain acts to reduce the activity of the signal-receiving neuron.

Ion channels: Pore-forming proteins that help establish and control the small voltage gradient across the plasma membrane of all living cells by allowing the flow of ions down their electrochemical gradient.

Ionotropic receptors: Receptors for neurotransmitters that act as ion channels. Binding of the neurotransmitter to the receptor changes the receptor’s structure so that the channel opens and ions can pass into or out of the cell.

Kinase: An enzyme that transfers phosphate groups from one molecule (the donor) to a specific target molecule (the substrate).

Ligand: Any substance that binds to a receptor.

Lipoprotein: A compound molecule that contains both a protein and a lipid component.

Long-term depression: Long-lasting mechanism contributing to neuroplasticity whereby an episode of ineffective or uncoordinated signal transmission at a synapse leads to a decreased effectiveness of subsequent signal transmission across that synapse; opposite of long-term potentiation.

Long-term potentiation: Long-lasting mechanism contributing to neuroplasticity whereby an episode of strong receptor activation at a synapse leads to a subsequent strengthening of the signal transmission across that synapse (e.g., by inducing the accumulation of more receptor molecules at that synapse).

Mesolimbic dopamine system: System of interconnected brain regions consisting of the ventral tegmental area, nucleus accumbens, and components of the limbic system, such as the amygdala; is considered the brain’s reward pathway that mediates the rewarding effects associated with alcohol and other drug use as well as other experiences.

Messenger RNA (mRNA): Key intermediary molecules that are generated during gene expression.

Meta-analysis: An analysis that combines the results of several studies addressing a set of related research hypotheses.

Metabotropic receptors: Receptors for neurotransmitters that indirectly lead to the opening of ion channels via second messengers. Binding of the neurotransmitter to the receptor activates G-proteins, which in turn induce the production of second messengers such as cyclic AMP (cAMP) that then activate kinases, ultimately resulting in the opening of ion channels in the cell’s membrane.

Metaplasticity: Concept whereby the neuroplasticity of a synapse is influenced by previous history of activity of that synapse.

Microglia: Type of glial cell that acts as an active immune defense in the central nervous system.

Myelin: A white fatty material composed chiefly of alternating layers of lipids and lipoproteins that encloses the axons of myelinated nerve fibers.

Myelinolysis: Dissolution of the myelin sheaths of nerve fibers.

Neuroactive steroid: Any of a group of steroid molecules that are naturally produced in the adrenal glands, ovaries, and testes and which can act on neurons and modulate their functions.

Neurogenesis: The process by which neurons are created.

Neuropeptide: Protein-like molecules made in the brain. Neuropeptides consist of short chains of amino acids, with some functioning as neurotransmitters and some functioning as hormones.

Neuroplasticity: Ability of the nervous system to change and reorganize itself throughout life by forming new connections among nerve cells or altering the activities of existing nerve cells and connections; is the basis for the ability to learn throughout life.

Neurotransmitter: Signaling molecules produced in nerve cells (neurons) that serve to transmit signals from one neuron to another neuron or from a neuron to another type of cell (e.g., muscle cell); neurotransmitters are released from the signal-emitting neuron and bind to receptors on the surface of the signal-receiving cell.

Nucleotide: A subunit of DNA and RNA.

Nucleus accumbens (NAc): A nucleus located in the forebrain that plays an important role in reward, pleasure, addiction, and fear; primarily contains GABAergic neurons).

Nucleus/nuclei: In neuroanatomy, a nucleus is a central nervous system structure that acts as a hub or transit point for electrical signals in a single neural subsystem.

Oligodendrocytes: A variety of glial cells whose main function is to insulate the axons in the central nervous system of the higher vertebrates.

Oscillation: The repetitive variation, typically in time, of a parameter about a central value (e.g., by a pendulum).

Parietal lobe: Part of the brain that is located at the top and to the sides of the brain, behind the frontal lobe; integrates sensory information from different organs, determines spatial sense and navigation.

Phenotype: The observable structural or functional characteristics of an individual organism that result from the interaction of its genotype with environmental factors.

Phosphatase: An enzyme that removes a phosphate group from a molecule.

Pons: A structure located on the brain stem. It relays sensory information between the cerebellum and cerebrum.

Posttranscriptional modification: The process by which precursor messenger RNA (mRNA) is converted into mature mRNA. This process is vital for the correct translation of the genomes and for the export of the mRNA from the nucleus for translation.

Posttranslational modification: The chemical modification of a protein after its translation; extends the range of functions of the protein by attaching to it other biochemical functional groups or by changing the chemical nature of an amino acid.

Presynaptic terminal: A specialized area within the axon of the presynaptic cell that contains neurotransmitters enclosed in small membrane-bound spheres called synaptic vesicles; also known as synaptic bouton.

Putamen: A round structure located at the base of the forebrain. The putamen and caudate nucleus together form the dorsal striatum. It also is one of the structures in the basal ganglia.

Receptor: A protein molecule located in the membrane surrounding a cell or in the cell’s interior that can bind with a signaling molecule (e.g., a hormone or neurotransmitter), thereby setting off a chain of biochemical reactions in the cell that alter the cell’s behavior in a specific manner.

RNA (ribonucleic acid): Macromolecule related in structure to DNA; typically a single-stranded molecule. Different types of RNA molecules are found in human cells and cells of other organism (e.g., messenger RNA [mRNA]).

Second messenger: A signaling molecule that participates in the intracellular reactions resulting when a stimulus, such as a neurotransmitter, binds to a receptor on the cell surface.

Selective serotonin reuptake inhibitors: A class of antide-pressants that increase the extracellular level of the neurotransmitter serotonin by inhibiting its reuptake into the presynaptic cell, increasing the level of serotonin available to bind to the postsynaptic receptor.

Scaffolding protein: A protein whose main function is to bring other proteins together for them to interact.

Single-nucleotide polymorphism (SNP): A DNA sequence variation occurring when a single nucleotide in the genome (or other shared sequence) differs between members of a species (or between paired chromosomes in an individual)

Splenium: The thickened posterior border of the corpus callosum.

Striatum: The major input station of the basal ganglia system. The striatum is made up of the caudate nucleus and the putamen.

Sulci: Large furrows that divide the brain into lobes.

Synapse: Region where two nerve cells interact to transmit nerve signals from one cell to the other.

Synaptic bouton: A button-like swelling on an axon where it has a synapse with another neuron; also known as axon terminal, presynaptic terminal, or synaptic knob.

Synaptic strength: Measure of the overall electrical signal that is generated in a postsynaptic cell at a synapse in response to the interactions of a neurotransmitter with its receptor(s) at that synapse.

Thalamus: A large, dual-lobed mass of gray-matter cells located at the top of the brainstem. It receives auditory, somatosensory, and visual sensory signals and relays sensory signals to the cerebral cortex.

Transcription factor: A protein that regulates gene expression by binding to specific sites on the DNA near the start of the gene.

Transcription: RNA synthesis, or transcription, is the process of transcribing DNA nucleotide sequence information into RNA sequence information.

Transcriptome: The entirety of all transcription products (transcripts or messenger RNA [mRNA]) present in a cell, tissue, or organism.

Transcriptosome complex: The complex of molecules that is involved in transcription.

Translation: The production of proteins by decoding messenger RNA (mRNA) produced in transcription.

Ventral tegmental area: Area located in the midbrain that contains dopaminergic nerve cell bodies that project to various parts of the forebrain, including the nucleus accumbens and other parts of the mesolimbic dopamine system.

Ventricles: Fluid-filled spaces in the brain.

Voltage-gated channel: Ion channel whose opening is controlled by changes in electrical charge (i.e., the electric potential measures in volts) that exist between the cell’s interior and exterior.

